# A Case of Severe Multisystem Inflammatory Syndrome in Children (MIS-C) Treated with Multiple Biologics

**DOI:** 10.1155/2022/6181922

**Published:** 2022-08-08

**Authors:** Beenish Zulfiqar, Hira Imran, Kathleen Collins

**Affiliations:** University of Tennessee Memphis, Department of Rheumatology, Memphis, USA

## Abstract

The COVID-19 virus has impacted global health on a wide scale, affecting humans of all ages and ethnicities. While most have mild upper respiratory viral symptoms, some have died due to severe pneumonia, acute respiratory distress syndrome (ARDS), or coagulopathies to mention a few. It has been postulated that the COVID-19 virus can initiate an autoinflammatory reaction in the body via multiple pathways of cytokine activation. The virus can cause delayed response after 4–8 weeks of acute infection, which resembles a cytokine storm or MAS (macrophage activation syndrome). This highly inflammatory syndrome, called MIS-C or multisystem inflammatory response syndrome, needs to be diagnosed and treated early to prevent multiorgan damage and mortality. There are widespread lab abnormalities including highly elevated acute phase reactants ferritin, D-Dimer, lactate dehydrogenase (LDH), creatinine kinase (CK), sedimentation rate (ESR), and C-reactive protein (CRP) as well as markers of cardiac damage including troponin and brain natriuretic peptide (BNP). The syndrome can present in unique ways from classic MIS-C with hypovolemic shock to Kawasaki disease-like presentation. We present a case of a 12-year-old boy who presented to Le Bonheur Children's Hospital in Memphis with classic signs and symptoms of “severe” MIS-C requiring intubation, multiple pressors, ECMO, and renal replacement therapy. He was treated successfully with immunomodulating medicines including intravenous immune globulin (IVIG), steroids, interleukin-6 inhibitor, tumor necrosis factor-a inhibitor, interleukin-1 inhibitor, and Janus kinase inhibitor.

## 1. Introduction

As defined by the US Centers for Disease Control and Prevention, MIS-C needs the following criteria for definition: serious illness leading to hospitalization, an age of less than 21 years, fever (body temperature, >38.0°C) or report of subjective fever lasting at least 24 hours, laboratory evidence of inflammation, multisystem organ involvement (i.e., involving at least two systems), and laboratory-confirmed SARS-CoV-2 infection (positive SARS-CoV-2 real-time reverse-transcriptase polymerase chain reaction (RT-PCR) or antibody test during hospitalization) or an epidemiologic link to a person with COVID-19 [1]. MIS-C tends to present 4–6 weeks after acute COVID-19 infection [2]. The number of documented symptomatic COVID-19 infections reported in children has been significantly less than that in adults due to milder forms of the disease. However, a certain population of children suffers from moderate to severe forms requiring hospitalization and critical care support [3]. In a systematic review published by Ahmed et al. in July 2020 [4], they reviewed 39 articles with a total sample size of 662 children with MIS-C. Children had widespread systemic involvement and more than 50% had Kawasaki disease (KD) overlap-like phenotypic features including rash and conjunctivitis. Various organ systems seem to be involved, most commonly the heart presenting with a low ejection fraction (45%). In comparing children with MIS with a milder presentation of COVID-19, MIS children had a much higher percentage of intensive care unit admissions (71% Vs 3.3%) and mechanical ventilation (22% to 0.54%). Hence, this condition requires an early diagnosis and prompt treatment for good overall outcomes.

## 2. Case

A 12-year-old boy (mixed race: Caucasian and African American) with a medical history of obesity, BMI (body mass index) 32.5, and asthma presented to our emergency department with 4-day history of high-grade fevers, vomiting, diarrhea, abdominal pain, loss of taste and smell, and decreased oral intake. He tested negative on PCR for COVID-19 and PCR for streptococcal throat; 6 weeks before presentation, his parents were infected with COVID-19, and our patient had a viral illness around that same time, although he was tested three times for COVID-19 about 6 weeks prior when his parents were tested positive, and all 3 times, he was tested negative for COVID-19 on PCR. On physical examination, he was tachycardic (heart rate: 120) and tachypneic (respiratory rate: 33). His temperature was 104 Fahrenheit, oxygen saturation was 100% on room air, and blood pressure was 120/53. Labs were significant for elevated inflammatory markers (see Table 1) (erythrocyte sedimentation rate >130 mm/hr, C-reactive protein: 272 mg/L, fibrinogen: 880 mg/dL, D-dimer: 6.78 mcg FEU/mL, procalcitonin: 11.27 ng/mL, and ferritin: 775 ng/mL). White cell counts were elevated at 11,000, and platelets were low at 101,000 thou/mcl. An electrocardiogram showed sinus tachycardia, and brain natriuretic peptide (BNP) and troponins were within normal limits. An echocardiogram showed trivial pericardial effusion and mild ectasia of the left main coronary artery measuring 5.1 mm. Total COVID antibodies (IgM + IgG) were positive. Given his history, abnormal vital signs, and elevated inflammatory markers with positive COVID antibodies, he was diagnosed with multisystem inflammatory syndrome (MIS-C).

**Figure 1:**
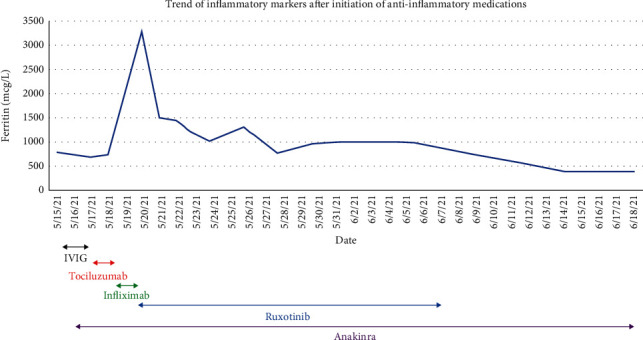
Ferritin trend against biologics initiation.

He was started on intravenous methylprednisolone 100 mg every 12 hours, subcutaneous anakinra 100 mg every 6 hours on day 1, and broad-spectrum antibiotics. The next day his inflammatory markers were further elevated, and his renal function worsened leading to renal failure. He eventually was intubated and developed persistent shock despite being on three pressors and inotropic support. He was then started on ECMO and continuous renal replacement therapy (CRRT). The medications were escalated to anakinra 100 mg every 4 hours, 1000 mg pulse dose of intravenous methylprednisolone for 5 days, 3 doses of 800 mg of intravenous tocilizumab, 2 doses of intravenous infliximab (500 mg and 300 mg), and oral ruxolitinib 20 mg every 12 hours and then once daily for 2 weeks (see Table 2). He was hemodynamically stable and taken off the ECMO in 5 days. The inflammatory markers started trending down during the first week and were in their normal range by the end of the treatment which lasted for a month (see Figure 1). The steroids were gradually tapered over a month, and anakinra was the last immunosuppressant to be discontinued. His MIS-C resolved, but unfortunately, the patient developed intracranial hemorrhage with multifocal areas of cerebral hemorrhage and left uncal herniation due to the anticoagulation used during ECMO and had to undergo decompression craniotomy. Physical and occupational therapy services evaluated the patient and deemed him appropriate for prolonged rehabilitation due to intensive care unit-acquired weakness, and hence he was discharged to an acute inpatient rehab facility.

## 3. Discussion

COVID-19 or SARS-CoV-2 is transmitted between humans mainly by respiratory secretions and via droplets on contaminated surfaces. It has been postulated that theoretically, SARS-CoV-2 enters the body of the host via angiotensin-converting enzyme 2 (ACE2) which is particularly abundant in alveolar cells, vascular endothelium, and in small amounts, immune cells [5]. SARS-CoV-2 generates an accelerated and exaggerated immune response in certain human hosts which in many ways can resemble cytokine storms. As we are aware that macrophage activation syndrome is a form of secondary hemophagocytic lymphohistiocytosis (HLH) linked to rheumatological conditions or malignancies, HLH has been specifically well studied regarding its pathophysiology and immunopathology over many decades. The major driver of the pathogenesis of HLH is a malfunction of natural killer cells and dysregulation of cytolytic CD8 cells (CTLs) leading to activation of antigen-presenting cells [6]. The excessive release of interferon-gamma (IFN-y) and TNF-a causes the recruitment of neutrophils and lymphocytes causing the release of pro-inflammatory cytokines such as TNF-a, IL-6, IL-1, and IL-18. A similar pathway is observed in patients with severe COVID-19 with a hyperinflammatory response [7].

There have been a few theories postulated to describe why hyperinflammation due to MIS-C tends to occur. Lucas et al. from Yale School of Medicine talked about two possible etiologies. The first one describes a rare second hit with a pathogen within a certain frame of time after getting the initial infection while the second one talks about a unique immune response to the virus triggering autoimmunity [8].

It is interesting to note that low platelets could be an indicator of MIS-C. In viral-associated hyperinflammatory syndromes (e.g., MIS-C), mediators are being secreted in the process of eradication of the virus, mainly to stimulate CD8+ cells to kill virally infected cells, which would inadvertently suppress bone marrow function and activate platelets, culminating in thrombocytopenia. This could be a useful distinguishing feature from other conditions like acute infection and vasculitis-like conditions such as Kawasaki disease [9].

In a case reported by Buonsenso et al. [10] in August 2020, they measured cytokine levels in a patient with a known diagnosis of MIS-C. The patient was found to have elevated levels of pro-inflammatory cytokines including IL-6, IL-1B, and TNF-a.

Cron et al. proposed that MIS-C should be included under the same theory and horizon of a cytokine storm. He described the effects of MIS-C on immunological levels causing lymphopenia, thrombocytopenia, and elevated serum IFN-*γ*, IL-1*β*, IL-6, IL-10, and IL-17 concentrations, although not to the same extent as in the more severe cytokine storm syndromes [11]. Our patient had a severe case of MIS-C which required prompt diagnosis and treatment. Our approach was to cater to and inhibit the major drivers of cytokine storm syndrome, namely, IL-1, IL6, TNF-a, and IFN gamma. Although we did not check for cytokines in blood since it is not routinely done, our goal was to recognize the storm early and to treat it effectively.

## Figures and Tables

**Table 1:** Laboratory values on admission.

Inflammatory marker	Value on admission	Reference range
CRP	270 mg/L	<9 mg/L
ESR	49 mm/hr	0–13 mm/hr
Absolute lymphocyte count (ALC)	1.08 thou/mcl	1.45–7.67 thou/mcl
Platelets	101 thou/mcl	140–450 thou/ml
Sodium	144 mmol/l	134–143 mmol/L
Neutrophils	35 thou/mcl	1.8–7.1 thou/mcl
Albumin	2.4 g/dl	3.5–5.4 g/dl
Ferritin	730 ng/ml	10–300 ng/ml
D-Dimer	4.50 cg-FEU/ml	0–0.44 mcg-FEU/ml
Fibrinogen	842 mg/dl	181–446 mg/dl
Hemoglobin	12.5	13.9 g/dL
ALT	56 U/L	10–41 U/L
INR	1.48	0.85–1.16
BNP	666.7 pg/ml	<100 pg/ml
Lactic acid	3.97 mmol/L	0.70–4.01 mmol/L

**Table 2:** Timeline and duration of anti-inflammatory drugs.

Medication	Date started	Date ended
IVIG	05/16/2021	05/17/2021
Anakinra	05/16/2021	06/23/2021
Infliximab	05/19/2021	05/20/2021
Tocilizumab	05/18/2021	05/19/2021
Ruxolitinib	05/20/2021	06/07/2021
SoluMEDROL	05/16/2021	06/25/2021

## Abbreviations

MIS-C: Multisystem inflammatory syndrome in children
ECMO: Extracorporeal membrane oxygenation
PCR: Polymerase chain reaction
IL: Interleukin
TNF-a: Tumor necrosis inhibitor-alpha
INF: Interferon
COVID-19: Coronavirus disease 2019
SARS-CoV-2: Severe acute respiratory syndrome coronavirus 2.

## Additional Points

*Key Messages.* (1) Although COVID-19 is not as common in children as in adults, the emerging mutants affecting all age groups are concerning. Hence, COVID-19 should be taken seriously in children. (2) Children diagnosed with COVID-19, even if they have mild symptoms or are asymptomatic, should be kept a close eye on for the development of cytokine storm like syndromes 4–8 weeks after acute infection. (3) A child presenting to the emergency room with an MIS-C type picture should be identified and started on treatment as soon as possible. Involvement of multiple disciplines like rheumatology, infectious disease, and cardiology should be done as soon as the child is admitted. (4) The treatment should be escalated without any delay. Like in our case, the child continued to deteriorate while on intravenous immunoglobulins (IVIG), intravenous steroids, and anakinra requiring the addition of other biologics.

## References

[1] Centers for Disease Control and Prevention (2019). Emergency preparedness and response: multisystem inflammatory syndrome in children (MIS-C) associated with coronavirus disease 2019 (COVID-19). https://emergency.cdc.gov/han/2020/han00432.asp.

[2] Feldstein L. R., Rose E. B., Horwitz S. M. (2020). Multisystem inflammatory syndrome in U.S. children and adolescents. *The New England Journal of Medicine*.

[3] WHO (2022). Multisystem Inflammatory Syndrome in Children and Adolescents with COVID-19. https://www.who.int/publications/i/item/multisystem-inflammatory-syndrome-inchildren-and-adolescents-with-covid-19.

[4] Ahmed M., Advani S., Moreira A. (2020). Multisystem inflammatory syndrome in children: a systematic review. *EClinicalMedicine*.

[5] Hamming I., Timens W., Bulthuis M., Lely A., Navis G. J., van Goor H. (2004). Tissue distribution of ACE2 protein, the functional receptor for SARS coronavirus. A first step in understanding SARS pathogenesis. *The Journal of Pathology*.

[6] Shimabukuro-Vornhagen A., Godel P., Subklewe M. (2018). Cytokine release syndrome. *Journal for ImmunoTherapy of Cancer*.

[7] Vastert S. J., van Wijk R., D’Urbano L. E. (2010). Mutations in the perforin gene can be linked to macrophage activation syndrome in patients with systemic-onset juvenile idiopathic arthritis. *Rheumatology*.

[8] Ramaswamy A. (2020). Nina B Post-infectious inflammatory disease in MIS-C features elevated cytotoxicity signatures and autoreactivity that correlates with severity. *medRxiv*.

[9] Yeo W. S., Ng Q. X. (2020). Distinguishing between typical Kawasaki disease and multisystem inflammatory syndrome in children (MIS-C) associated with SARS-CoV-2. *Medical Hypotheses*.

[10] Buonsenso D., Di Sante G., Sali M., Gabriele M. D., Sali M. (2020). Cytokine profile in an adolescent with pediatric multisystem inflammatory syndrome temporally related to COVID-19. *The Pediatric Infectious Disease Journal*.

[11] Henderson L. A., Canna S. W., Schulert G. S. (2020). On the alert for cytokine storm: immunopathology in COVID-19. *Arthritis & Rheumatology*.

